# Gut Instincts: Knowledge, Attitudes, and Practices regarding Soil-Transmitted Helminths in Rural China

**DOI:** 10.1371/journal.pntd.0003643

**Published:** 2015-03-25

**Authors:** Louise Lu, Chengfang Liu, Linxiu Zhang, Alexis Medina, Scott Smith, Scott Rozelle

**Affiliations:** 1 Rural Education Action Program, Freeman Spogli Institute for International Studies, Stanford University, Stanford, California, United States of America; 2 Center for Chinese Agricultural Policy, Institute of Geographical Sciences and Natural Resources Research, Chinese Academy of Sciences, Beijing, China; University of Queensland, AUSTRALIA

## Abstract

**Background:**

Soil-transmitted helminth (STH) infections affect more than two out of every five schoolchildren in the poorest regions of rural China, an alarmingly high prevalence rate given the low cost and wide availability of safe and effective deworming treatment. Understanding of local knowledge, attitudes, and practices regarding STH infection in rural China has until now, been sparse, although such information is critical for prevention and control initiatives.

**Methodology/Principal Findings:**

This study aims to elucidate the structural and sociocultural factors that underlie high STH infection rates as well as explain *why* deworming treatment is rarely sought for children. In-depth, qualitative interviews were conducted in six rural villages in Guizhou Province; participants included schoolchildren, children’s parents and grandparents, and village doctors. Data analysis exposed three predominant reasons for high STH prevalence: (1) lack of awareness and skepticism about the high prevalence of STH infection, (2) local myths about STH infection and deworming treatment, and (3) poor quality of village health care.

**Conclusions/Significance:**

The findings from this study reveal reasons for why deworming treatment is not sought, and inform specific recommendations for a deworming intervention that can more effectively address underlying barriers to deworming in areas of persistently high STH infection rates. The main barrier to seeking STH treatment is not availability or cost of the drugs, but rather the lack of impetus to seek the drugs. A comprehensive nationwide deworming program in China should involve annual provision of free deworming treatment in village clinics or schools, distribution of culturally appropriate educational materials to inform children and families about STH infection, and improvement of the quality of health care delivered by village clinicians.

## Author Summary

Soil-transmitted helminths (STHs) are parasitic intestinal worms that infect more than two out of every five schoolchildren in rural China, an alarmingly high prevalence given the low cost and wide availability of safe and effective deworming treatment. Understanding of local knowledge, attitudes, and practices regarding STHs in rural China has until now, been sparse, although such information is critical for prevention and control initiatives. This study elucidates the structural and sociocultural factors that explain *why* deworming treatment is rarely sought for schoolchildren in poor villages of rural China with persistently high intestinal worm infection rates. In-depth, qualitative interviews were conducted in six rural villages in Guizhou Province; participants included schoolchildren, children’s parents and grandparents, and village doctors. We found evidence of three predominant reasons for high STH prevalence: lack of awareness and skepticism about STHs, local myths about STHs and deworming treatment, and poor quality of village health care. The findings have significant relevance for the development of an effective deworming program in China as well as improvement of the quality of health care at the village level.

## Introduction

Soil-transmitted helminths (STH) are a group of parasitic intestinal worms that can infect humans through ingestion of parasite eggs or skin contact with motile larvae. Four STH species are of particular significance in public health: roundworm (*Ascaris lumbricoides*), whipworm (*Trichuris trichiura*), and two species of hookworm (*Necator americanus* and *Ancylostoma duodenale*) [1]. As of June 2013, it was estimated that more than one billion people around the world are infected with at least one of these four species [2].

Infections with *Ascaris lumbricoides*, hookworm and whipworm are often asymptomatic, but can cause consequences such as anemia and other nutritional deficiencies, stunted growth, and cognitive impairment in the long run [3]. Infections with *A*. *lumbricoides* can cause abdominal pain, lactose intolerance, and decreased absorption of vitamin A and other nutrients. Severe infection with whipworm can cause inflammation at the site of attachment in the intestines and result in colitis and rectal prolapse. Infection with hookworm may lead to intestinal blood loss that results in iron-deficiency anemia [4]. Chronic and intense STH infections contribute to malnutrition and other significant consequences for growth and cognitive development, primarily in children [4, 5]. The diverse and non-specific nature of the clinical manifestations of STH infection hinders patients and even clinicians from making a definitive diagnosis without a stool sample.

Anti-helminthic drugs can effectively treat STH infections in afflicted individuals as well as be utilized in mass drug administration among populations of children living in STH-endemic areas [4]. Albendazole, the most common pharmaceutical treatment used to treat STH infection, is a broad spectrum antihelminthic with high cure rates and fecal egg count, although efficacy varies among the different STH species. A review of seven trials testing the efficacy of albendazole among infected schoolchildren in different endemic areas found cure rates of 98.2%, 87.8%, and 46.6% for *A*. *lumbricoides*, hookworm, and *T*. *trichiura* respectively, and fecal egg count (FEC) reductions of 99.5%, 94.8%, and 50.8% for *A*. *lumbricoides*, hookworm, and *T*. *trichiura* respectively [6]. Side effects from albendazole are minor and relatively rare, and it is safe for use in mass drug administration programs [7]. The low cost of albendazole facilitates its wide availability in many developing countries, including large developing countries such as China and India. This in turn increases the cost-effectiveness of large-scale deworming initiatives.

Historically, STH infections have been a longstanding public health challenge in China. In the 1950s and 60s, the Chinese government recognized the problem and took steps to control high infection rates, integrating STH control measures into the rural health care system [8]. Unfortunately, such health measures have since been discontinued, and the problem of STH infection has re-emerged with evidence of increased prevalence over the past several decades, especially in impoverished and remote communities [9].

Recent studies conducted in China have highlighted both high STH prevalence as well as disparities in infection prevalence between rural and urban areas [10]. In 2010, researchers at Stanford University, the Chinese Academy of Sciences, and the Chinese Center for Disease Control and Prevention found that over 40 percent of school-aged children in poor regions of rural Guizhou Province were infected with at least one of the four most common STH species [9]. These high rates of infection were confirmed by a follow-up survey in 2013 conducted by the same research team [5].

Given the evidence of high STH infection rates in China—where safe, effective, and affordable treatment is available—the goal of this qualitative research study is to understand *why* there is such a high prevalence of these infections. In this paper, we investigate knowledge, attitudes, and practices regarding STHs in rural China to reveal the sociocultural and structural factors underlying the persistently high infection prevalence. We also seek to understand why so few individuals in rural communities seek deworming treatment. We anticipate that the findings will be valuable for informing the design and implementation of effective deworming campaigns in China as well as broader public health efforts.

## Methods

### Ethics Statement

This research was approved by the Human Subjects Committee at Stanford University and by the appropriate authorities at the Chinese Centers for Disease Control and Prevention. All relevant research procedures adhered to the guidelines of both institutions. Prior to conducting the interviews, we ensured that the participants understood the information given in the written consent process, which was reviewed and approved by the ethics committee of the Institutional Review Board at Stanford University in Stanford, California (Protocol ID 25027), as well as the Institutional Review Board at Sichuan University in Chengdu, China (Protocol ID 2013005–02). All study participants received treatment for STH infection at the conclusion of the study.

### Study Location

The study was conducted in Guizhou Province, located on the eastern part of the Yunnan-Guizhou Plateau in southwest China. Guizhou's population, estimated to be about 35 million in 2011, is demographically diverse with ethnic minority groups making up more than 37 percent of the population [11]. Guizhou Province is the second poorest of China’s 31 provinces, with most people living in extremely remote mountainous areas that lack adequate road infrastructure. Agriculture is the main occupation of most Guizhou inhabitants, and farmers in Guizhou often live in poverty with poor access to basic health services [11].

**Fig 1:**
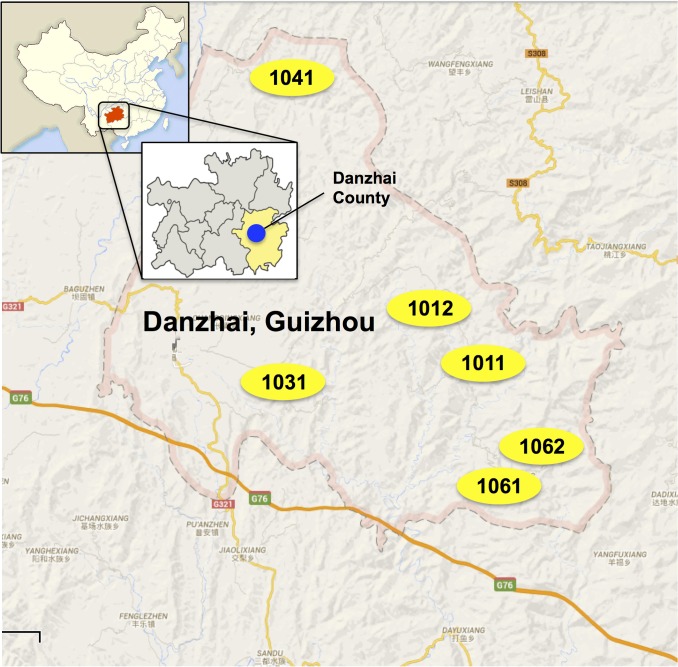
Map of the study area and location of the six villages in Danzhai, Guizhou, China.

The study area included six villages in four different townships in rural Guizhou Province (*Fig. 1. Map of the study area and location of the six villages in Danzhai, Guizhou, China)*. Villages within Guizhou Province were chosen for this study based on inclusion in the research team's ongoing randomized controlled trial and accessibility over several days to conduct the fieldwork [5].

### Participants

In total, 49 interviews were conducted: 23 elders, 23 children, and three village doctors. The elders were either parents or grandparents of the children who participated in our study. Children in each village were chosen randomly, with their ages ranging from 9 to 12 years old. Among the children, 12 were boys and 11 were girls. In the six villages that we visited, twenty households were of the Miao minority ethnic group while three were of the Shui minority ethnic group.

STH infection status (positive or negative) was determined at the laboratory of the county CDC using the Kato-Katz smear method on stool samples collected from schoolchildren. The study team collected one stool sample per day from each child for two consecutive days; two smears were taken from each stool sample for testing. Children were considered positive for STH infection if either one of their stool samples tested positive for one or more types of STH. Village level infection rates ranged from 13 to 57 percent (*Table 1. STH infection prevalence by village in Danzhai County, Guizhou)*.

**Table 1:** STH infection prevalence by village in Danzhai County, Guizhou.

***Village code***	***Infection Prevalence*** * ***(%)***
1011	13
1012	20
1031	25
1041	45
1061	57
1062	38

* Percentage of schoolchildren infected with at least one of three STH types.

In our study sample of 23 children, 8 tested positive for infection with at least one of the four STH species (roundworm *Ascaris lumbricoides*, whipworm *Trichuris trichiura*, and hookworm *Necator americanus* and *Ancylostoma duodenale*) and 15 tested negative for infection. The researchers were blind to the children's infection status at the time of the interview.

### Data Collection

Qualitative methodology for this study was comprised of in-person interviews based on semi-structured questionnaires. The interview questions consisted of an extensive list of open-ended questions that prompted individuals on various themes related to STH infection and treatment. The interview guide began with obtaining initial demographic information about the family members. The questions then progressed into inquiries about knowledge, attitudes, and practices regarding STHs and deworming treatment.

The interview protocol questions varied depending on whether the interviewee was a child, a parent/grandparent of a child, or a village doctor. The most extensive interviews were conducted with the parents and grandparents of the children, with several open-ended questions surrounding four topics: children's overall health and hygiene; opinions about the village doctor; knowledge about STH infection, prevalence, and health consequences of infection with STHs; and attitudes and practices regarding seeking of deworming treatment. Questions for the children focused on their knowledge, or lack of knowledge, about STHs. Questions for the village doctors aimed to assess the extent to which they were knowledgeable about STHs, whether deworming treatment was available in the clinic, and how often they prescribed deworming treatment to patients.

The interviews were conducted in both Mandarin Chinese and the Guizhou dialect of Chinese. One member of the research team in the field was a native of Guizhou Province, and clarified translations to the rest of the team. The interview protocol served as the primary guide, and it was ensured that all sections were covered. Some flexibility was utilized in following up on interviewees’ statements with additional probes and questions. Participants in the study were not paid monetarily, but each household surveyed was given a small gift of a hand towel (valued at around 3 USD) in gratitude for their participation.

### Data Analysis

Extensive field notes and transcriptions of audio-recorded interviews were closely analyzed using the constant comparative method described by Strauss and Corbin, in which codes from the data were constantly being compared to other identified codes to elucidate similarities, differences, and patterns in our collected data [12]. The coding process consisted of several readings, after which an initial process of open coding was performed on the detailed field-notes and transcripts, followed by focused coding, in which we extracted subcodes that emerged from prominent or recurring themes, trends, and ideas in the data.

Interview data from parents, children, and village doctors were also supplemented with memos and field observations in the households and villages. Thus, analyses from multiple data sources were synthesized to formulate the most accurate and complete representation possible of the knowledge, attitudes, and practices regarding STH infection and deworming treatment in rural Guizhou.

## Results

Three predominant reasons for high STH infection prevalence emerged from analysis of the data: lack of awareness about STHs; local myths about worms and deworming, and poor quality of village health care.

### Lack of Awareness about STHs. Reason One: Skepticism of High STH Prevalence

Parents and grandparents are both unaware and highly skeptical of the fact that STH infection is common in children today. In response to inquiries about how children become infected with STH, the most common response was “I don't know.” *(Table 2. Frequency of Household Interview Responses)*

**Table 2:** Frequency of household interview responses.

		***# Households***
**Skepticism of High Worm Prevalence**	Response of "*no"* to the question: "*Do you think your child has worms*?"**	22 of 23 (96%)
**Skepticism of High Worm Prevalence**	Response of "*none" or "one"* to the question: "*For example*, *there are five children from the village are playing together; how many would you guess have worm infection*?"**	20 of 23 (87%)
**Deworming of Piglets**	Response of "*yes"* to the question: "*Do you deworm your pigs*?"**	18 of 18* (100%)
**Distant Parent-Child Relations**	Number of households in which one or more parents migrated to the city for work	16 of 23 (70%)

*We were informed during Interview #6 of 23 that deworming of pigs was a universal practice in the village. Thus, we began explicitly asking this question in every subsequent household interview, totaling 18 responses.

A few parents seemed to have a slightly better recognition of possible transmission, responding that perhaps children become infected when they eat unclean, raw foods, or when they play outside in the fields and get their hands and feet soiled.

"*Kids probably get worms from eating things that are not clean*. *Also*, *from getting dirty while playing …" [Father of Child 106208*, *Positive STH Infection]*

It should be noted that these responses were still quite vague, and seemed more like guesses than informed statements of known fact. Moreover, from the interview questions regarding health and hygiene practices in the household, all children admitted to drinking water straight from the tap and going barefoot while playing both indoors and outdoors. While some of the elders recognized that these behaviors could potentially be linked to risk factors for STH infection, they were not proactive about discouraging these behaviors among their children.

In addition, some parents and grandparents noted their perceived symptoms of STH infection, which included loss of appetite, yellow face, and teeth-grinding.

"*If my grand daughter had worms*, *she would probably have a yellow or pale face*."* [Grandfather of Child 104104*, *Positive STH Infection]*

"*A kid with worms would have no appetite so he would be very skinny*. *Also*, *he would grind his teeth at night*. *And his stomach would hurt*."* [Father of Child 106105*, *Negative STH Infection]*

"*Children with worms*…*they would have no appetite*. *Their skin and their face would be yellow*. "*[Grandfather of Child 103121*, *Negative STH Infection]*

Similar to the elders' statements about STH transmission, these responses likewise seemed to be vague guesses about physical manifestations linked to general "sickness" rather than informed statements or observations corresponding to STH infection.

When asked whether they thought that their children were currently infected with STH, most parents responded with confident denial.

"*No*, *she definitely does not have worms right now*. *If she did*, *her stomach would be hurting*."* [Father of Child 106106*, *Positive STH Infection]*

"*I do not think my child has worms*. *She does not seem sick*."* [Father of Child 106104*, *Positive STH Infection]*

When asked to approximate the prevalence of STH in the village by estimating how many children out of a randomly chosen group of five village children would be infected, the majority of the parents and grandparents responded that it would be unlikely that any of the children would have STH infection.

"*Worms are not common in this village*. *Worms are not common at all these days*. *Out of five people*? *Zero would have worms*."* [Father of Child 106208*, *Positive STH Infection; From Village 1062 with 38% STH Infection Rate]*

However, when asked to estimate the frequency of STH infection in the villages when *they* were young children, the parents responded that most, if not all, of their peers back then were likely to have been infected with STH. In fact, many of them recalled having STH infections themselves. They believe that STH infections are a disease of the past because the quality of living conditions in the village has improved since their childhood years.

“*Back when I was a child*, *everybody had worms*. *During that time*, *out of five children*…*all of them would have worms*.” *[Father of Child 104107*, *Negative STH Infection]*

"*Worms were more common back then*. *People are eating better food now and the houses are nicer*."* [Grandmother of Child 105118*, *Negative STH Infection]*

When asked to consider their reactions to the hypothetical situation that their child was in fact, infected with STH, the parents responded without hesitation that they would be very concerned. They stated that their response would be to take their child to the doctor and that they would want their child to be treated.

“*If someone said that my daughter had worms*, *I would definitely worry*. *I would quickly take her to the doctor and get her checked*.”* [Mother of Child 106213*, *Positive STH Infection]*

The responses from the parents about how they would respond if they knew that their child contrasted with the reality of the situation. The problem is that the parents and grandparents have little to no knowledge about STH infection and are highly skeptical that their children could possibly be infected. The knowledge gap serves as the primary barrier towards the seeking of deworming treatment.

#### Deworming of piglets

Another significant discovery was that all of the families interviewed remarked that they regularly deworm their piglets. They believe that STHs are very common in pigs and view regular deworming as a ubiquitous and necessary practice for their piglets to grow normally.

"*Of course we deworm our piglets*. *Pigs have lots of worms*. *They eat all kinds of vegetables and things on the ground*. *Everyone in the village does this*. *They would be silly if they do not*."* [Mother of Child 104113*, *Negative STH Infection]*

When asked about the symptoms of STH infection in pigs, their responses were similar to what they said would be symptoms of STH infection in children (i.e. lack of appetite, "skinny-ness").

"*The seller of pig feed tells everyone to give [deworming] medicine to our pigs*. *We get a free package [of deworming treatment] when we buy pig feed*. *He told us that it helps our pigs grow fat*. *So even when our small pigs look normal*, *we still give them the medicine so that they can grow big*."* [Grandfather of Child 103121*, *Negative STH Infection]*

"*We deworm the piglets once every other month*. *The seller of feed told us to do that*."* [Grandfather of Child 105119*, *Positive STH Infection]*

The parents believe that it is important to deworm their piglets, thereby resulting in the pervasive practice of regular deworming of piglets in the villages.

#### Distant parent-child relations

In rural China, it is common for parents to leave their farming villages when their children are very young in order to seek better wages in the cities. Termed the "left-behind children" phenomenon, this situation was apparent in the villages that we visited in rural Guizhou. In sixteen of the twenty-three households included in our study sample, one or both parents had left the village to seek employment in an urban area. When both parents were absent from the household, the grandparents were the primary caretakers of the children.

Analysis of conversations with children, grandparents, and the few parents who remained in the villages reveals various reasons why parent absenteeism may have an influence on the seeking of intestinal worm treatment.

"*Grandparents just make sure that kids are fed well*. *Grandparents don't care much about sanitation or about worms or other things that you said*. *Moms and dads*, *like me*, *we pay more attention to more things about our child's health*…*but lots of parents are out of the home*."* [Mother of Child 104106*, *Negative STH Infection]*

One reason is that adults in the grandparents' generation have lower levels of schooling, on average, than members of the parents' generation. It was significantly more common for grandparents to have undergone no schooling at all, while most parents had completed a few years of primary school education. A lack of education among the grandparents may translate into less awareness and knowledge about STH, as well as less initiative in seeking health care services. Members of the parents' generation expressed a recognizable willingness to adopt new ideas as well as internalize recommendations for behavior changes, an attribute that was less characteristic of the grandparents' generation. For example, we interviewed a mother who remarked that she regularly gave deworming treatment to her son after her township doctor recommended it to her.

"*When my son was two years old*…*the doctor*, *the doctor in the township*, *told me that my son should take deworming medicine every six months*. *So I listened to his words and gave my son a white pill until he was about eight years old*."* [Mother of Child 105120*, *Positive STH Infection]*

### Reason Two: Local Myths about STH and Treatment

#### Myth: STHs are essential for digestion

We observed an intriguing belief that was pervasive among households in multiple villages, the belief that STHs are critical to one's health and that every person—from children to adults to the elderly—harbors some STHs.

"*Everyone has worms*! *Because if you did not have any worms*, *then you would not be able to digest your food*. *Everyone knows this*. *Some worms are small and some are big*. *The only problem is when the worms get big*."* [Father of Child 105104*, *Negative STH Infection]*

The idea that STHs are a natural and essential component of digestive health significantly detracts from the desire to seek deworming medicine. If one believed that STHs were necessary for one's health, it would seem preposterous to intentionally deworm a child regularly, especially when the child is in his or her growth and development stages.

*My teacher told me when I was young [that worms are essential for good health]*. *Yes*, *the child's mother knows this too*. *Everyone knows this*, *or they should know it*."* [Grandfather of Child 101110*, *Negative STH Infection]*

This idea initially seemed contradictory to the simultaneous belief among elders that STH infection is rare among children in the villages, so we asked for clarification during the interview. The elders explained that the two ideas were not mutually exclusive. They believe that everybody has worms because worms are essential for digestion. *However*, it can become a health issue if the worms become "very big", but that this problematic situation is not common at all in children today.

As part of the randomized controlled trial from which we obtained our study population, children who identified as positive for STH infection were offered treatment (two 200 mg tablets of albendazole, to be taken at home) and were instructed to relay information about the disease and the treatment to their parents or grandparents. When parents and grandparents were asked about the administration of the deworming medicine, however, many reported giving their children only one of the two albendazole tablets, if they followed instructions at all.

As part of the deworming medicine distribution process to the children in the randomized controlled trial, the dosage was specifically designated as two pills (equivalent to 400 mg) of albendazole—the amount recommended by the World Health Organization based on epidemiological surveys and literature reviews. Thus, adults who received deworming medicine but prevented their child from taking the full 400 mg dose (two pills) in order to "*only get rid of some worms*, *but not all of them"* were actually failing to treat the child’s worms.

#### Myth: Deworming medicine hurts fertility

Interviews revealed another myth regarding STHs that was popular among adults in the villages—particularly grandparents over sixty years old. The belief was that deworming treatment has a negative effect on children's future fertility. Members of both the parents’ generation and grandparents’ generation expressed this belief:

*It is known that deworming medicine has bad effects*…*it can reduce fertility in people who take it*."* [Grandfather of Child 104107*, *Negative STH Infection]*

Evidently, the topic of fertility was a sensitive one for elders in rural villages. After receiving the response from the grandfather quoted above, we modified our interview protocol to include culturally sensitive questions that could indirectly prompt our interviewees to mention this particular myth, if relevant to their beliefs. We discovered that this belief was not unique to a single household or a single village; it was a belief shared by many adults in rural Guizhou.

We perused the medical literature in an attempt to uncover the origins of the belief, but the notion that deworming medicine administered to children harms their future fertility has no scientific backing and is absent from medical literature. We also looked at the anthropological literature with no success. Indeed, the origins of the deworming and fertility myths are unclear. The negative biases about STH treatment among the elderly population in rural Guizhou, however, appear to play a critical role in preventing grandparents from seeking or accepting deworming medicine for children.

### Reason Three: Poor Quality of Village Health Care

#### Absence of the village doctor

We sought out the village doctor as our first point of contact in the villages that we visited; however, out of the six villages that we visited, only three had doctors who were present, highlighting the lack of availability of the village doctors in rural Guizhou.

Only one of the three village doctors had a designated clinic area and had seen any patients within the past month. The other two village doctors were untrained individuals who were assigned by upper-level township officials to assume the position.

Many villagers we interviewed responded that their village did not have a village doctor, or that if there was a village doctor, they did not know who he or she was and would not consider seeing him or her for care.

"*Village doctor*? *We do not have one*…*perhaps there is a doctor but I do not know who he is*. *It has been many years since there was a doctor in the village*. *If we need a doctor*, *we go to the township*."* [Father of Child 101102*, *Negative STH Infection]*

With the absence of a reliable village doctor, families must seek treatment at the next level: the township health center. Traveling to the township health center by bus or minivan costs both money and time. Depending on the remoteness of the village, the township health center could be anywhere from thirty minutes to several hours away. The cost of medical care at the township center is also considerably more expensive than within the villages.

#### Unavailable and misinformed doctors

Among villages that had available village doctors, the health care provided was little better or more reliable than at villages without village doctors. The village doctors that we interviewed were both under-informed and misinformed about STH prevalence and treatment.

“*No one has come in asking about worm medicine in the past half year*. *Worms are not common in kids anymore*. *My kid has never had worms*. *If a kid has worms*, *they will have a pale or yellow face and have a stomachache*…*and won't be eating*.”* [Village Doctor 103; From Village 1031 with 25% STH Infection Rate]*

In another village, the designated doctor was a young adult male who had been assigned the role more than a year ago.

"*There used to be a different village doctor here before me*, *but people here never went to see him*. *They just went to the township hospital*. *So the village doctor migrated out to work*. *I was assigned to take his place because it is required*. *I don't have medical experience*. *Local people here never come to me so I would prefer to go find work in the township too*."* [Village Doctor 106; From Village 1061 with 57% STH Infection Rate]*

Although the village doctor admitted to having no medical background to be able to reliably diagnose illnesses if patients were to consult with him, he responded that he did carry albendazole in his clinic. He had no means of performing a stool examination in the clinic to test for soil-transmitted helminths, but he said that if parents were to request the deworming medicine, he would be able to provide the treatment for 2 RMB (about 30 cents USD).

In another village, the doctor could not be found in her designated clinic area, but we interviewed her in one of the households that we visited. She mentioned that parents hardly ever ask her about STH infection in children. However, she also claimed to carry albendazole and said that she would provide it to parents for 1 RMB per pill (about 15 cents USD) if they requested the medicine.

*[Village Doctor 104; From Village 1041 with 45% STH Infection Rate]*: *Yes*, *I have albendazole*. *I sell them by the pill*, *for one kuai each*. *But this is only for adults*.

*[Interviewer]*: *What is only for adults*?

*[Village Doctor]*: *Albendazole*. *It is only for adults because it is bad for young kids*. *I can start giving it to kids when they are around twelve or thirteen years old*.

*[Interviewer]*: *Where did you first hear that*?

*[Village Doctor]*: *Nobody had to tell me*. *Me*, *I know this*. *It is from my experience*.

*[Interviewer]*: *So in the past*, *did you ever give albendazole to kids*? *How did they*, *or their parents*, *respond*?

*[Village Doctor]*: *Yes*. *So this was not good*. *Parents came back saying*, "*there were no worms in my kids' stool*!"* And I would tell them*, "*Sorry*, *maybe your kid did not have any worms*."**

Evidently, the village doctor’s knowledge of worms and deworming treatment was limited and misinformed. She supplied albendazole to parents in the form of single 200 mg tablets, only half of the WHO-recommended dose of 400 mg. In addition, her statement that albendazole is "bad for young kids" is inaccurate, as the safety and efficacy of albendazole in children of preschool and school-age has been extensively confirmed in the medical literature.

## Discussion

Field interviews with parents, grandparents, children, and village doctors revealed insight into the knowledge, attitudes, and practices surrounding STH in rural China. The three major reasons identified in the *Results* section provide a comprehensive understanding of the sociocultural and structural factors that appear to contribute to high STH prevalence among schoolchildren in rural Guizhou.

First, a lack of knowledge among parents and grandparents about intestinal STH infection, prevalence, and prevention precludes them from seeking deworming treatment for their children. Elders are unlikely to suspect that their children are infected, primarily due to widespread skepticism about the high prevalence of STH infection in villages today, a paradigm that hinders them from seeking deworming treatment for their children.

Many elders stated that STH infections were much more common when they were children; their belief that STH infections are a problem of the past contributes to their skepticism that their child could possibly be infected. One possible reason for this belief might have been that the intensity of worm infection was higher among the population when the parents were children—this might have led to more obvious symptoms. Due to the very sparse data that is available regarding STH infection from past decades, however, such speculations remain unproven. Parents and grandparents seemed fairly candid about discussing the commonality of having worms in their villages when they were young, and even about having had worms themselves. Therefore, it seems that stigma did not create significant bias, and that elders were truly unaware of the possibility that their child had STH infection. Interestingly, knowledge that STH are common in piglets is widespread, and deworming of piglets was performed on a regular basis among all of the households in our study.

Second, myths about STH infections and deworming treatment, such as the false beliefs that STH are essential to people's digestion and that deworming medicine harms children's future fertility, serve as barriers to the idea that children will benefit from improved health upon receiving deworming medicine. Such myths were more common among older generations; this impacts children directly because grandparents have become the primary caretakers of the majority of the children in rural China today due to the phenomenon of left-behind children.

Third, the absence and overall unreliability of local doctors in the villages—who themselves have faulty knowledge about STH infection, prevalence, and treatment—serve as the most direct barrier to the seeking of STH treatment. The lack of reliable access to knowledgeable medical professionals who could provide deworming medicine was an especially significant obstacle in remote villages that were located hours away from the township health center.

### Implications for Public Health and Policy

The results from this qualitative study supplement our understanding of the myriad factors that contribute to high STH prevalence in rural China. This information serves to inform health policymakers about how to approach and design more effective and comprehensive deworming interventions.

First, the finding that parents are skeptical of the high prevalence of STH infection among children in the villages points to the need for greater awareness and health education. Parents, grandparents, and village doctors were uninformed about STH prevention, manifestations, and treatment. The rural Chinese health care system recognized STH as a public health problem and prioritized regular deworming of children from the 1960s to the 1980s. The subsequent disappearance of STH from the national health agenda likely facilitated the notion among adult generations that STH infection has diminished or disappeared as a problem in children today. Redirecting attention towards the problem of STH infections in rural China may provide the necessary stimulus for individuals to realize and internalize the continued significance and seriousness of the problem.

The practice of regularly deworming piglets, which was common among all households interviewed, portends significant implications for a public health intervention. The majority of households in rural China raise at least one or two pigs each year as a critical source of income [13]. From the interviews, it seemed that parents practiced regular deworming of their piglets for two simple reasons: first, because they receive free deworming pills every time they purchase pig feed, and second, because they were told that the pills would produce faster-growing, healthier pigs. If village doctors were to consistently provide parents with free deworming pills twice a year, they could prompt an analogous paradigm shift in which parents make two realizations to encourage deworming as a pervasive practice: first, STH infection is common in children (as much or even more so than in piglets), and second, deworming leads to improved child health and growth.

The problem of distant parent-child relations preventing children from opening up to their caregivers about their health symptoms is rooted deeply in sociology and therefore, more challenging to address through health policy. However, the implication of this finding is that regularly administering deworming treatment to children in school or at home (by village doctors or trained local health personnel, like barefoot doctors) should be preferred to an alternative approach that relies on caregivers themselves to take the initiative to seek deworming treatment.

The discovery of myths pertaining to STH infection and deworming medicine (i.e. that helminths are necessary for digestion and that deworming medication harms fertility) presents an extra dimension to the lack of accurate knowledge about STH infection and treatment. The educational component of a comprehensive deworming intervention should actively debunk these myths in a culturally sensitive way while also presenting factual information about STH infections and the benefits of treatment. Parents and grandparents should be cautioned that their beliefs in myths about STHs and deworming treatment are unsupported by scientific and medical evidence, and may be preventing them from properly attending to their child's health.

In summary, an understanding of parents’ and grandparents’ attitudes towards STH and deworming treatment indicates that the main barrier to seeking treatment is not availability or cost of the drugs, but rather the lack of *impetus* to seek the drugs. A successful deworming intervention can be designed to work around this barrier, firstly by providing accurate information, but also by tasking village doctors or community health workers with administering albendazole in the households, rather than relying on compliance by the elders or children themselves.

Furthermore, witnessing the absence and poor reliability of village doctors in rural Guizhou highlights the need for improvement in the local health care system. The New Cooperative Medical System (NCMS) requires all villages to have a designated village doctor, hence the "assignment" of individuals in the community to be the village doctor in the cases where none is present [14]. Despite the NCMS requirement, we were unable to locate village doctors in three out of the six villages that we visited. Lack of access to an adequately trained village doctor and, by extension, to deworming treatment, serves as the final barrier to accessing treatment for children. Even when village doctors were present, they themselves were uninformed about STH infections and some did not carry albendazole in their clinic.

Greater attention should be directed towards structurally improving the state of the rural health care system in China as well as increasing the competence of local doctors. In addition, stricter oversight—by township and county officials—over village doctors and the medications that are stocked in their clinics would improve the reliability of doctors and encourage more individuals to seek health care at the local level.

### Researcher Effects and Limitations

The study sample was comprised of twenty-three households and three village clinicians in six villages across four townships in Guizhou Province. While we attempted to include villages that had variable characteristics in terms of population size, geographical location, density of households in the village, and proximity to the nearest township center, the results of this study may not be representative of all households across all of the different villages in rural Guizhou Province. However, the diverse sample of households provided a collection of perspectives to inform conclusions that can be reasonably applied to poor rural households in Guizhou Province.

While we do have information on prevalence of STH infection in the sample areas, we do not have information on the intensity of infection in these areas, which could have enlightened us as to whether infected individuals would be likely to have experienced acute symptoms.

Despite adherence to a carefully designed and meticulously reviewed interview protocol, qualitative interview data has the potential to be influenced by inherent limitations: recall bias, social desirability bias, and possibly even the intentional misinformation or withholding of information on behalf of the participants. We attempted to minimize social desirability bias and intentional misinformation as much as possible by phrasing our questions carefully. For example, we asked adults what they perceived the deworming practices of their neighbors to be, in addition to their own practices, in case families wished to protect their own practices (no significant distinction was noted). Moreover, when appropriate, all interviews were all conducted in the local dialect in an attempt to build trust between the research team and the families.

### Conclusions

The high prevalence of STH infections among millions of schoolchildren is a serious public health problem in rural China today. The findings of this study fill a critical gap in the current literature regarding the complex combination of factors that contribute to high STH burden in rural China despite the availability of effective and affordable treatment.

First and foremost, there is a lack of both knowledge about STH and awareness of high infection rates in the villages, which prevents parents and grandparents from suspecting the problem of STH infections in their children. In the rare case that caregivers *do* realize that children in the household have STH infections, myths and false local beliefs deter and discourage them from seeking deworming treatment. The final barrier lies in the inadequate village health care system, in which village doctors are absent from their clinics or are themselves misinformed about STH. This makes deworming medication difficult to obtain even when caregivers realize that their children are infected with STHs *and* are willing to seek treatment.

The results of this study suggest that a comprehensive deworming program to reduce STH infection rates in rural China should include the following components: free deworming treatment that is provided annually either through schools or village clinics, educational materials that provide accurate and necessary knowledge about STH, an emphasis on the impacts of deworming on children's educational attainment and future financial prospects, education about STH infections through community engagement, cultural sensitivity when overturning local myths about STH infections and deworming treatment, and lastly, greater efforts towards improving the quality of the village health care system.
