# Correction: Gut Instincts: Knowledge, Attitudes, and Practices regarding Soil-Transmitted Helminths in Rural China

**DOI:** 10.1371/journal.pntd.0003732

**Published:** 2015-04-23

**Authors:** Louise Lu, Chengfang Liu, Linxiu Zhang, Alexis Medina, Scott Smith, Scott Rozelle

There is an error in the second sentence of the third paragraph in the Introduction. The correct sentence is: Albendazole, the most common pharmaceutical treatment used to treat STH infection, is a broad spectrum antihelminthic with high cure rates and fecal egg count reductions, although efficacy varies among the different STH species.
